# Facile Green Synthesis of Ni(OH)_2_@Mn_3_O_4_ Cactus-Type Nanocomposite: Characterization and Cytotoxicity Properties

**DOI:** 10.3390/molecules27248703

**Published:** 2022-12-08

**Authors:** Amel Taha, Hanaa A. Hassanin

**Affiliations:** 1 Department of Chemistry, College of Science, King Faisal University, P.O. Box 400, Al-Ahsa 31982, Saudi Arabia; 2 Department of Chemistry, Faculty of Science and Technology, Al-Neelain University, Khartoum 11121, Sudan; 3 Department of Chemistry, Faculty of Science, Ain Shams University, Abbassia, Cairo 11566, Egypt

**Keywords:** Ni(OH)_2_@Mn_3_O_4_, nanocomposite, antitumor, chia seeds, MCF-7

## Abstract

In the present work, the facile eco-friendly synthesis and evaluation of the anti-tumor activity of Ni(OH)_2_@Mn_3_O_4_ nanocomposite were carried out. The synthesis of Ni(OH)_2_@Mn_3_O_4_ nanocomposite from chia-seed extract was mediated by sonication. The obtained materials were characterized by different spectroscopic techniques such as transmission electron microscopy (TEM), scanning electron microscope (SEM), energy-dispersive X-ray spectroscopy (EDS), X-ray diffraction (XRD), X-ray photoelectron spectroscopy (XPS), ultraviolet-visible (UV-Vis), and Fourier transform infrared (FT-IR) spectroscopies. The results of XRD, SEM, EDS, TEM, FT-IR, and UV-Vis analysis indicate the successful manufacturing of a crystalline, cactus-type Ni(OH)_2_@Mn_3_O_4_ nanocomposite of 10.10 nm average particle size. XPS analysis confirms that the synthesized materials consist mainly of Ni^2+^, Mn^2+^, and Mn^3+^. The antitumor activity of the nanocomposite was tested against a breast cancer (MCF-7) cell line. The results showed Ni(OH)_2_@Mn_3_O_4_ nanocomposite possesses insignificant cytotoxicity. The cell-death percentage was 34% at a 100 ppm concentration of Ni(OH)_2_@Mn_3_O_4_ nanocomposite. The obtained results imply that the synthesized nanocomposite could be suitable and safe for drug delivery and water treatment.

## 1. Introduction

Cancer is a disease caused by abnormal cell division and is considered one of the major worldwide health problems [1]. The common treatment methods that are used in cancer therapy are chemotherapy, radiation therapy, immunotherapy, surgery, targeted therapy, and hormone therapy [2,3]. Such methods are usually associated with serious side effects and an increased risk of recurrences. Therefore, nanoparticles have recently been used to overcome the limitations of conventional treatment methods [4]. Nano-drug carrier systems have shown advantages in cancer therapy due to the high penetration of nanoparticles into human tissue [5]. Nanotechnology and nanoscience are multidisciplinary fields that use unique methodologies and exceptional results to make use of the vast range of burgeoning fields and fundamental disciplines like physics, chemistry, electronics, and material science with novel techniques and produce nanomaterials with uncommon and unique properties on the nano scale [6].

Metal nanocomposites are multicomponent materials that consist of many phases; at least one has a nanoscale diameter [7]. They show numerous outstanding properties in comparison with their conventional counterparts. Nanocomposites are extensively employed in many fields such as drug delivery [8], water remediation [9,10,11], photocatalysis [5], supercapacitors [12,13,14,15] industrial catalysts [16], and recently in bio-medical applications as antibacterial and cancer treatments [17].

There are several forms of metal nanocomposites, including metal–metal, metal–metal hydroxide, metal–metal oxide, metal oxide—metal oxide, and metal–organic polymer. It is necessary to develop new classes of nanomaterial—nanoparticles (NPs)—and improve functionalities to overcome the limitation of mono-nanoparticles and enhance their properties [12,13].

Metal nanocomposites can be synthesized through chemical and physical routes. However, these methods have adverse effects on the environment and are not economically viable [18,19]. Hence, the growth of environmentally friendly routes for synthesizing metal nanocomposites without using hazardous materials is important [9]. This can be achieved through the use of plant extracts as reducing agents. Different parts of plants such as leaves, fruits, roots, stem, and seeds have been applied for producing various nanoparticles due to the presence of phytochemicals in their extracts, which act like stabilization and reducing agents [10]. 

Metal nanocomposites are amongst the nanomaterials that have been widely examined due to their wide-ranging availability in chemical sensors, microwave absorbers, permanent magnets, and in bio-medical applications like drug delivery and in cancer thermotherapy. They have shown significant pharmacokinetic advantages in cancer treatment; lately, they have been used as anticancer materials for different kinds of cancer cells such as (A549), HepG-2, and MCF-7 cell lines [14,15,16].

Mn_3_O_4_ is a mixed oxide material, and one of the important nanoparticles, which encompasses many crystal forms. Because it is naturally abundant, inexpensive, and exhibits a variety of oxidation states, it has been used extensively in different fields, including catalysis, electrochemistry, and medicinal chemistry [20,21,22,23]. Mn_3_O_4_ has improved biological capabilities and low toxicity, and it has the potential to be used in antibacterial systems due to its synergistic behavior [24]. Mn^2+^ generates non-toxic reactive species that have been widely applied in the treatments of many diseases such as diabetes, heart disease, and cancer. It is anticipated as a promising research area for chemotherapy [25].

Since Ni(OH)_2_ has diverse uses in many fields, is considered the most effective nanomaterial among other metal hydroxide nanomaterials. To a limited extent, Ni(OH)_2_-NPs have been characterized in vivo and in vitro applications [26]. According to a literature review, there are different methods for the synthesis of nanomaterials. These methods include the use of chemical solvents that have a negative influence on the environment. The green production of nanoparticles refers to the employment of plant extracts in the production of nanoparticles.

In nanocomposites, metal and metal oxide nanoparticles show different physiochemical characteristics. They have extremely exciting semiconducting capabilities and are highly recommended for the growth of biological activities [27]. For example, Mn_3_O_4_/Ni(OH)_2_ nanocomposite could be used as a practical electrode material for pseudo capacitors [14].

The current work aims to manufacture Ni(OH)_2_@Mn_3_O_4_ nanocomposite using chia-seed extract as an environmentally safe, cheap, and fast method to limit chemical consumption by substituting chemical stabilizing and capping agents with aqueous chia-seed extract assisted by ultrasound radiation. In addition, the antitumor behavior of the manufactured nanocomposite toward the MCF-7 cell line was examined.

## 2. Experimental and Methods

### 2.1. Materials

Light chia seeds were bought at a local market in Hufof, Saudi Arabia’s Eastern Province. Manganese (II) chloride, 97% (MnCl_2_); nickel (II) chloride hexahydrate, 99.9% (NiCl_2_·6H_2_O); and potassium hydroxide, ≥85.0 %, were obtained from Sigma-Aldrich (St. Louis, MO, USA). All solutions were made using deionized water.

#### 2.1.1. Chia Extract Preparation

To prepare the aqueous chia seed extract, 100 mL of deionized water was combined with 4.0 g of crushed seeds and heated to 80 °C for 15 min. The extract was next doubly filtered through a home sieve system and Whatman No. 1 filter paper. The extract was stored at 4 °C for later use.

#### 2.1.2. Synthesis of Nanomaterials

The green synthesis of Ni(OH)_2_@Mn_3_O_4_-NC was accomplished according to our earlier work for the synthesis of Ni(OH)_2_ [28]. In brief, 25 mL of 0.1 M metal chloride salts (NiCl_2_ and MnCl_2_) in a 1:1 ratio were mixed with 25.0 mL of aqueous chia extract with constant stirring at room temperature followed by dropwise addition of KOH (1.0 M) to fix the solution pH at a value of (11.0). The pH of the solution was recorded using a Thermo Fisher Scientific Orion 2 Star (Waltham, MA, USA) pH meter. After this, the sample solution was subjected to a 30-min sonication process using a Power-Sonic 405 (Hwashin, Korea) device with a working frequency of 40 kHz and a maximum input power of 350 W. The samples that had been prepared were then thoroughly rinsed with deionized water and allowed to dry at room temperature.

#### 2.1.3. Characterization

To examine the characteristics of the prepared nanoparticles various analytical techniques were used. A UV-Vis spectrophotometer (Shimadzu, Kyoto, Japan) was used to analyze the optical properties of produced materials. Dynamic light scattering (DLS) was recorded using a Cilas (Orléans, France) dual scattering particle size analyzer Nano DS. Fourier transform infrared spectroscopy (FT-IR) was collected using a (Cary, NC, USA, (630 FT-IR)) spectrophotometer. Field emission-scanning electron microscopy (FE-SEM) was recorded using a scanning electron microscope, model (FEI, QUANTA FEG, 250 high-resolution field emission electron microscope), coupled with a high-angle, angular dark-field detector, and an X-ray energy-dispersive spectroscopy system (EDX). Transmission electron microscopy (TEM) images were recorded using (JEOL JEM-2100, Tokyo, Japan) at an acceleration voltage of 90 KV. To examine the crystalline phase of the produced nanomaterials, X-ray diffraction spectroscopy (XRD) was carried out using an X-ray diffractometer (EMPYREAN by Cu Ka radiation with a wavelength of 1.54 °A). Debye–Scherrer’s equation was applied to calculate the nano-crystallite size from the width of the XRD peaks. The surface structure and the oxidation state of the manufactured samples were analyzed using X-ray photoelectron spectroscopy (XPS). K-ALPHA XPS instrument (Thermo Fisher Scientific, USA) aligned with monochromatic X-ray Al K-alpha radiation −10 to 1350 eV spot size 400 μm at pressure 10^−9^ mbar with full-spectrum pass energy 200 eV and at narrow-spectrum 50 eV was used to record the XPS spectra.

#### 2.1.4. In Vitro Antitumor Activity

The MCF-7 cell line was obtained from the American Type Culture Collection (Rockville, MD, USA). The tumor cells were then cultured in DMEM (Dulbecco’s Modified Eagle Medium) with 10% FBS (fetal bovine serum) and 100 U/mL of penicillin and streptomycin. The cells were grown at 37 °C in a humidified incubator with 5% CO_2_ [29].

The antitumor activity of the produced materials was assessed against the MCF7 cell line using an MTT assay. The MTT assay is based on tetrazolium salt cleavage by mitochondrial dehydrogenases in viable cells. The cells were placed in 96-well sterile microplates (5 × 10^4^ cells/well) and incubated at 37 °C with DMSO solutions of the test compounds for 48 h in a serum-free medium prior to the MTT assay. The negative control was considered as the medium culture free from the tested materials. The medium in each well was carefully removed and replaced with 40 μL of MTT (2.5 mg/mL) after incubation. The samples were then incubated for another 4 h. To dissolve the purple formazan dye crystals 200 μL of DMSO was added. Absorbance was then recorded at 570 nm using a SpectraMax Paradigm Multi-Mode microplate reader. The relative cell death was determined by calculating the mean percentage of dead cells relative to the control sample. All experiments were performed in triplicate on different days. All the values are reported as the mean ± SD. SPSS software (SPSS Inc., Chicago, IL, USA) was used to perform probit analysis to determine IC_50_ values.

### 2.2. Results and Discussions

#### 2.2.1. Characterization

##### UV-Vis Spectroscopy

**Figure 1:**
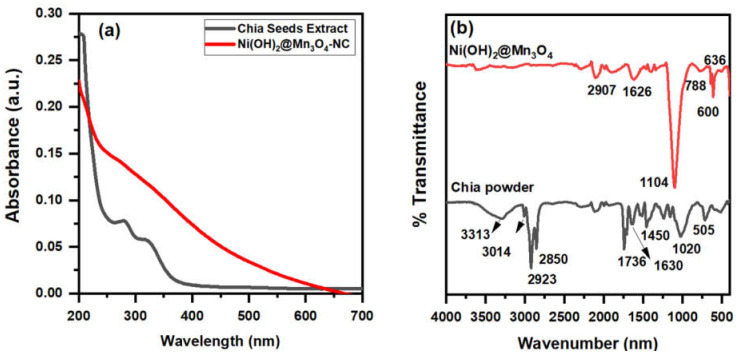
(**a**) FT-IR and (**b**) UV-Vis spectra for chia powder and Ni(OH)_2_@Mn_3_O_4_-NC.

UV-Vis Spectroscopy was utilized to examine the electronic characteristics of synthesized materials and chia-seed extract. Figure 1a. shows the absorption spectra for Ni(OH)_2_@Mn_3_O_4_-NC, and chia-seed extract in the range 200–700 nm. The absorption spectrum of the chia-seed extract shows peaks at 280 and 320 nm that are assigned to the C=O group of glucose and protein contents of chia-seed extract, respectively [30]. The Ni(OH)_2_@Mn_3_O_4_-NC UV-Vis spectrum shows a more linear spectrum in the range 250–350 nm and this could be because of the existence of Ni(OH)_2_, which decreases the light-illuminating properties of the nanocomposite [25,26].

##### FT-IR Spectroscopy

The FTIR spectra of chia-seed powder and their corresponding prepared nanocomposite are presented in Figure 1b. The appearance of absorption bands at 3200–3500 cm^−1^ is correlated to the stretching vibrations of -OH groups [31,32,33]. Additionally, the vibrational bands in the 1745–1460 cm^−1^ are assigned to (C=O) for the amide I groups in the protein content of chia seed. The methyl and methylene C-H stretching frequencies that appear at 2923–2850 cm^−1^ are related to the presence of lipids and proteins, respectively [27].

The FT-IR spectrum for the as-prepared nanocomposite exhibits small peaks in the range of 3200–3600 cm^−1^, which could be attributed to the vibrational mode of the hydroxyl group (O–H) of interlayer water molecules and the H-bonded -OH groups [28,29]. Ni(OH)_2_@Mn_3_O_4_-NC FT-IR spectrum reveals characteristic peaks at 1104, 788, 636, and 600 cm^−1^, which are characteristic for both Mn-OH, Mn-O, and Ni-OH, Ni-O vibrations indicating the successful synthesis of the Ni(OH)_2_@Mn_3_O_4_ nanocomposite [31]. The C-H stretching frequency of the methyl and methylene backbones of chia-seed lipids appears at 2907 and 1626 cm^−1^, respectively [34,35,36,37,38]. These results affirm the success of the green production of Ni(OH)_2_@Mn_3_O_4_ nanocomposite.

#### 2.2.2. FE-SEM and EDS Analysis

**Figure 2:**
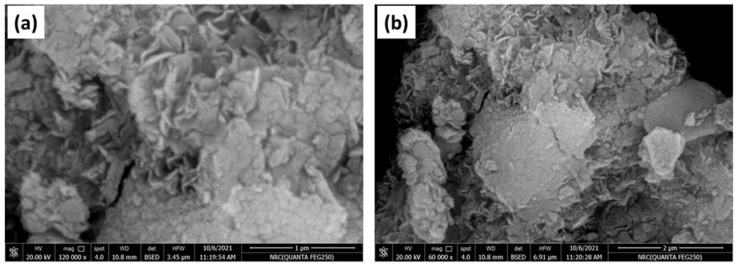

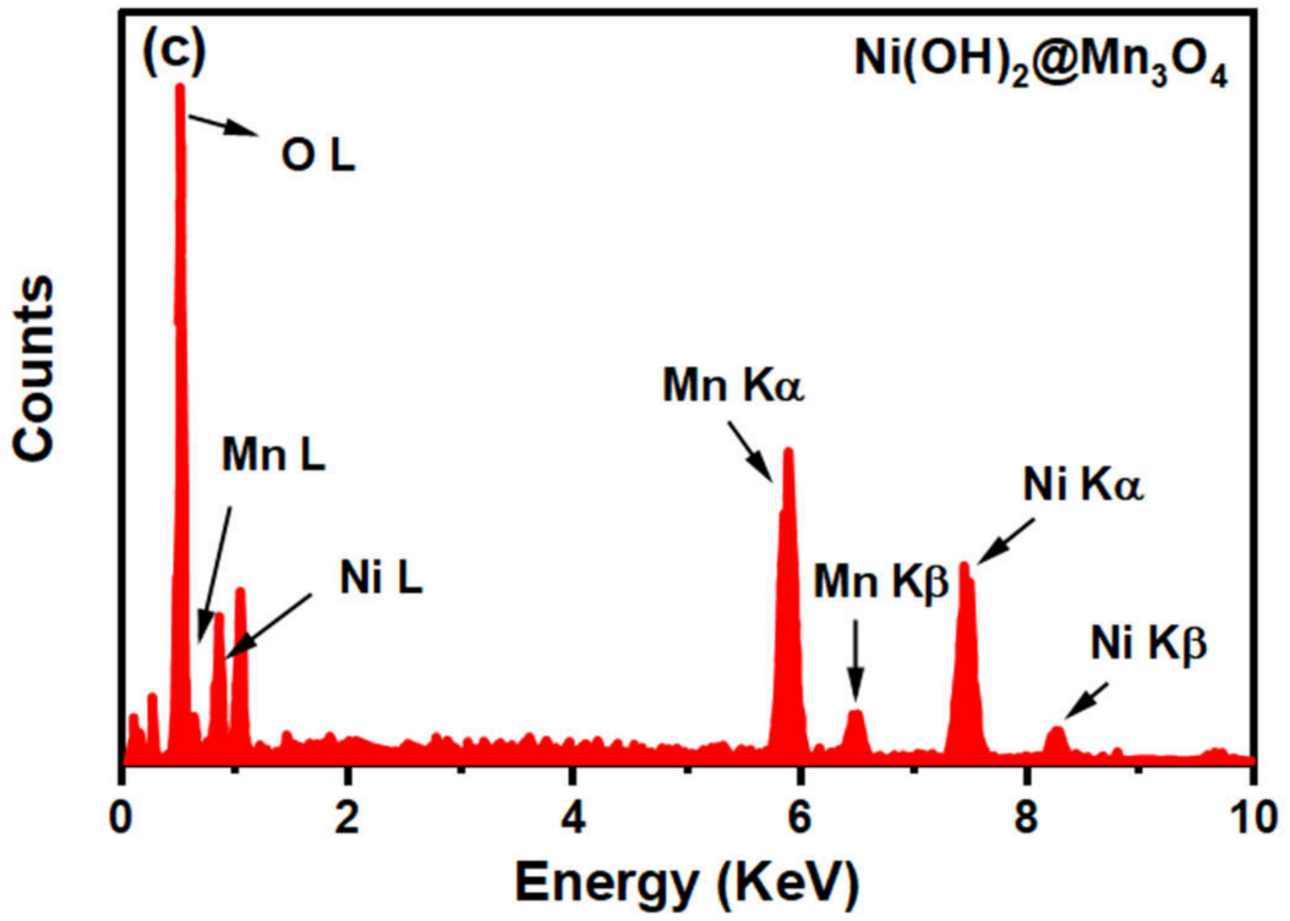
(**a**,**b**) FE-SEM images with different magnification scales (**c**) Energy Dispersive Spectroscopy (EDS) for Ni(OH)_2_@ Mn_3_O_4_ NC.

To examine the morphological and structural characteristics and elemental components of the obtained nanoparticles, field emission-scanning electron microscopy (FE-SEM) and X-ray energy dispersive spectroscopy (EDS) were performed on the prepared materials. The formed nanocomposite, Ni(OH)_2_@Mn_3_O_4_ shows agglomerated cactus-type nanoparticles morphology as shown in Figure 2a,b with different scales of 1 and 2 µm, respectively.

Figure 2c illustrates the EDS microanalysis of Ni(OH)_2_@Mn_3_O_4_ nanocomposite. The results show the existence of Ni, Mn, and O components solely in the obtained nanocomposite, indicating the successful synthesis of pure Ni(OH)_2_@Mn_3_O_4_ nanocomposite [39,40,41].

#### 2.2.3. HR-TEM and DLS Analysis

**Figure 3:**
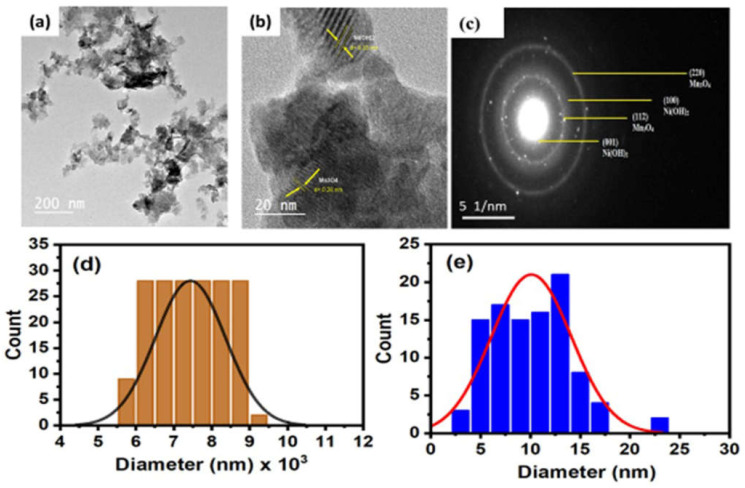
(**a**) TEM image (**b**) HR-TEM image of the composite with lattice fringes (**c**) Selected electron area diffraction pattern (SEAD), Particle size distribution calculated from (**c**) DLS analysis, (**d**) TEM analysis for Ni(OH)_2_@Mn_3_O_4_-NC and (**e**) Particle size distribution from TEM for Ni(OH)_2_@Mn_3_O_4_-NC.

The manufactured samples’ size and morphology were examined using HR-TEM analysis. The HR-TEM image for the Ni(OH)_2_@Mn_3_O_4_ nanocomposite is presented in Figure 3a. The images show that the nanocomposite is agglomerated irregular shape particles mixed with thin hexagonal segments due to the existence of Ni(OH)_2_ [42]. Figure 3b shows the (001) and (112) crystal planes of the Ni(OH)_2_ and Mn_3_O_4_ in which the lattice fringes could be matched with d-spacings 0.35 nm and 0.28 nm, correlated with the phase of theophrasite hexagonal crystal system of Ni(OH)_2_ (#014-0117) and the phase of tetragonal hausmannite Mn_3_O_4_ (#24-0734) [43]. The SEAD (selected area of electron diffraction) pattern of synthesized Ni(OH)_2_@Mn_3_O_4_ nanocomposite is presented in Figure 3c and clearly shows the multilayered patterns, confirming the polycrystalline nature of the green synthesized nanocomposite.

The prepared nanocomposite’s particle size distribution from the dynamic light scattering (DLS) investigation is shown in Figure 3d. The mean particle diameter is (~7430 nm) for Ni(OH)_2_@Mn_3_O_4_ as calculated from DLS, whereas the grain-size distribution estimated from TEM analysis is 10.1 nm using Image J software as represented in Figure 3e. The difference between the particle sizes measured by DLS and TEM analyses could be explained by the existence of layers of organic molecules from plant extract linked to the nanocomposite. Due to their electron transparency, these organic compounds do not appear in TEM [44]. These results indicate the manufacturing of Ni(OH)_2_@Mn_3_O_4_-NC using chia extract as a stabilizing and capping agent [45].

#### 2.2.4. XRD Analysis

**Figure 4:**
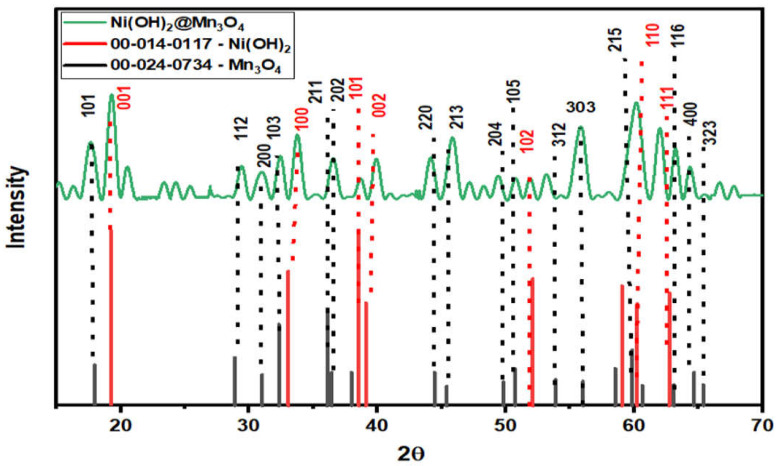
XRD diffraction pattern of Ni(OH)_2_@Mn_3_O_4_-NC.

The crystal phase of the synthesized nanomaterials was tested using XRD. The XRD pattern of Ni(OH)_2_@ Mn_3_O_4_-NC aligned with the respective JCPDS pdf card, as shown in Figure 4. The XRD profile of the obtained nanocomposite (Ni(OH)_2_@Mn_3_O_4_) reveals distinctive peaks of both Mn_3_O_4_ and Ni(OH)_2_. the diffraction plans (001), (100), (101), (002), (102), and (110) are related to the principal peaks at 2θ values of 19.2°, 33.1°, 38.5°, 39.0°, 52.1°, and 59.5°, respectively. This matches the reference pattern of theophrasite hexagonal crystal system of Ni(OH)_2_ according to pdf card number # 014-0117 [46,47,48,49]. The peaks at 18.0°, 29.0°, 32.2°, 36.4°, 56°, 5°, 60.4°, and 65.3° correspond to the (101), (112), (102), (202), (220), (303), (215), and (323) diffraction plans of tetragonal hausmannite Mn_3_O_4_ (JCPDS 24-0734) [46] which proves the successful production of Ni(OH)_2_@Mn_3_O_4_ nanocomposite [13,47].

The particle diameter of the as-prepared nanocomposite was calculated by the Scherrer equation [50]. The mean crystallite size calculated using the Scherrer equation was found to be 11.5 nm for Ni(OH)_2_@Mn_3_O_4_-NC, which agrees well with the mean diameter calculated from TEM measurements.

##### X-ray Photoelectron Spectroscopy (XPS)

**Figure 5:**
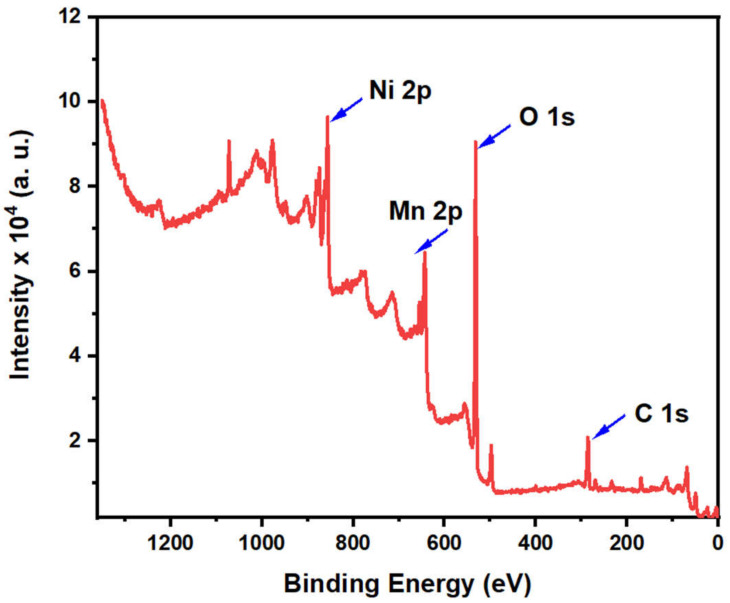
X-ray photoelectron survey scan spectrum for the synthesized Ni(OH)_2_@Mn_3_O_4_ nanocomposites.

**Figure 6:**
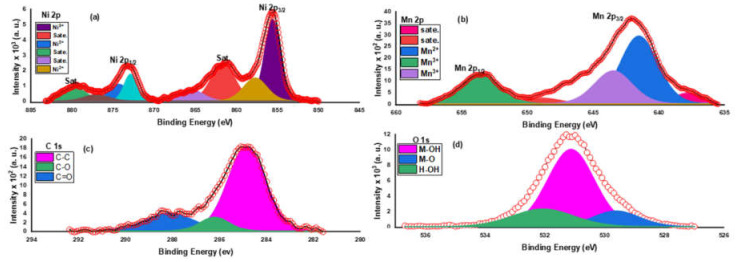
XPS spectra of (**a**) Ni (2p) (**b**) Mn 2p (**c**) C (1s) (**d**) O (1s) spectra for the produced Ni(OH)_2_@Mn_3_O_4_ nanocomposite.

XPS, X-ray photoelectron spectroscopy analysis, was used to examine the surface structure and the oxidation state of the synthesized samples. The survey scan of the prepared materials indicated the purity of the samples; hence it contains only M, O, and C elements, where M represents Ni and/or Mn. Figure 5 illustrates the survey scan of the Ni(OH)_2_@Mn_3_O_4_ nanocomposite. The survey spectrum shows that the elements Ni, Mn, O, and C exist without any other contaminants, suggesting the successful synthesis of the nanocomposite. Figure 6a represents the high-resolution spectrum of Ni 2p. The Ni 2p spectrum consists of two shakeup satellites and two main peaks at 855.5 and 873.3 eV that correspond to Ni 2p_3/2_ and Ni 2p_1/2_ spin-orbit peaks, respectively, which indicates the existence of Ni(OH)_2_ [51]. The high-resolution spectrum of Mn 2p (Figure 6b) consists of two main peaks at 641.98 and 653.5 eV, which are attributed to Mn 2p_3/2_ and Mn 2p_1/2_ orbits, respectively [52].

The C (1s) spectrum can be split into three principal peaks which are related to C-C, C-O, and C=O, respectively (Figure 6c) [53]. The XPS spectrum of O 1s for all prepared samples reveals the presence of characteristic peaks that are related to M-O-M, surface hydroxide groups, and adsorbed water molecules, respectively (Figure 6d) [54]. The co-existence of C in nanomaterials could be attributed to the chia extract used in the green synthesis of the nanomaterial [55]. Table 1 shows FWHM, atomic percentages, and the binding energy of all peaks for the produced materials.

#### 2.2.5. Cytotoxicity Assessment for the Synthesized Samples

The antitumor efficiency of the produced Ni(OH)_2_@Mn_3_O_4_ nanocomposite toward the MCF-7 cell line was examined using MTT assay. It has been previously reported that Ni(OH)_2_ has a potential activity toward several cancer cell lines (83.2%) [28]; however, it has been reported that Mn_3_O_4_ nanoparticles have been less toxic to some kinds of cell lines such as HT29 cells [56] and L929 fibroblast cells [57]. The results showed that the manufactured Ni(OH)_2_@Mn_3_O_4_ nanocomposite has insignificant cytotoxic activity toward the examined cell line. The cell mortality was found to be 34% at 100 ppm concentration of Ni(OH)_2_@Mn_3_O_4_. The lower cytotoxic behavior of Ni(OH)_2_@Mn_3_O_4_ nanocomposite could be attributed to the presence of Mn_3_O_4_-NP in the produced nanocomposite. These results suggest that produced Ni(OH)_2_@Mn_3_O_4_ nanocomposites could be safe to use for water remediation and drug delivery [58].

## 3. Conclusions

Ni(OH)_2_@Mn_3_O_4_ nanocomposite was successfully synthesized via an environmentally safe, easy, and cheap method using aqueous chia-seed extract. FT-IR confirmed the interaction between different function groups in the chia-seed extract and the synthesized nanocomposite. XRD analysis confirmed the crystalline structure of the nanocomposite and investigated the size in the range of 11 nm. SEM showed agglomerated cactus-type nanoparticle morphology. According to the XPS survey spectrum, the produced nanocomposite consists of the elements Ni, Mn, O, and C existing without any other contaminants, suggesting the successful synthesis of the nanocomposite. The antitumor activity of the manufactured nanocomposite was tested against a breast cancer (MCF-7) cell line. The results showed that the Ni(OH)_2_@Mn_3_O_4_ nanocomposite has insignificant cytotoxicity toward the cancer cells, with a cell mortality of 34% at 100 ppm concentration. Based on a literature review, Mn_3_O_4_ nanoparticles showed lower toxicity toward some cancer cell lines. Therefore, the lower cytotoxic activity of Ni(OH)_2_@Mn_3_O_4_-NC could be attributed to the presence of Mn_3_O_4_-NP.

## Figures and Tables

**Table 1:** FWHM, atomic percentages, and the binding energy (eV) for the prepared samples.

Sample Name		Binding Energy (eV)	FWHM	Atomic %
**Ni(OH)_2_@ Mn_3_O_4_**	O 1s	531.91	3.54	49.18
	Ni 2p	856.42	4.34	15.11
	Mn 2p	643	5.9	13.21
	C 1s	285.96	3.98	22.7

## Data Availability

Data only available upon request from the corresponding author.

## Sample Availability

Samples of the compounds are available from the authors.
